# Impact of Metabolic Regulators on the Expression of the Obesity Associated Genes *FTO* and *NAMPT* in Human Preadipocytes and Adipocytes

**DOI:** 10.1371/journal.pone.0019526

**Published:** 2011-06-08

**Authors:** Daniela Friebe, Dennis Löffler, Maria Schönberg, Falk Bernhard, Petra Büttner, Kathrin Landgraf, Wieland Kiess, Antje Körner

**Affiliations:** 1 University Hospital for Children and Adolescents, University of Leipzig, Leipzig, Germany; 2 Leipzig University Medical Center, IFB Adiposity Diseases, Leipzig, Germany; University of Las Palmas de Gran Canaria, Spain

## Abstract

**Background:**

FTO and NAMPT/PBEF/visfatin are thought to play a role in obesity but their transcriptional regulation in adipocytes is not fully understood. In this study, we evaluated the transcriptional regulation of *FTO* and *NAMPT* in preadipocytes and adipocytes by metabolic regulators.

**Methodology and Principal Findings:**

We assessed *FTO* mRNA expression during human adipocyte differentiation of Simpson-Golabi-Behmel syndrome (SGBS) cells and primary subcutaneous preadipocytes *in vitro* and evaluated the effect of the metabolic regulators glucose, insulin, dexamethasone, IGF-1 and isoproterenol on *FTO* and *NAMPT* mRNA expression in SGBS preadipocytes and adipocytes. *FTO* mRNA levels were not significantly modulated during adipocyte differentiation. Also, metabolic regulators had no impact on *FTO* expression in preadipocytes or adipocytes. In SGBS preadipocytes *NAMPT* expression was more than 3fold induced by dexamethasone and isoproterenol and 1.6fold by dexamethasone in adipocytes. Complete glucose restriction caused an increase in *NAMPT* mRNA expression by more than 5fold and 1.4fold in SGBS preadipocytes and adipocytes, respectively.

**Conclusion:**

*FTO* mRNA expression is not significantly affected by differentiation or metabolic regulators in human adipocytes. The stimulation of *NAMPT* expression by dexamethasone, isoproterenol and complete glucose restriction may indicate a regulation of NAMPT by metabolic stress, which was more pronounced in preadipocytes compared to mature adipocytes.

## Introduction

Previously, various genes including the *fat mass* and *obesity associated* (*FTO*) and the *nicotinamide phosphoribosyltransferase* (*NAMPT*) gene have been suggested to potentially contribute to the development of obesity and related metabolic traits [1], [2]. Several independent genetic association studies have associated variants in the *FTO* gene with predisposition to childhood and adult obesity [1], [3], [4]. The location of the variants in intron 1 of the *FTO* gene may indicate a putative effect on gene transcription regulation. Of interest, *FTO* variant rs8050136, located ∼270-kb proximal to FTO [5], was demonstrated to influence retinoblastoma-like 2 *(RBL2)* gene expression. RBL2 is a key regulator of entry into cell division that may also play a role in adipogenesis [6]. *FTO* is widely expressed in human fetal and adult tissues, particularly the brain [1]. In addition, expression in metabolically active tissues such as skeletal muscle, liver and adipose tissue has been reported [7], [8]. It has also been demonstrated that inactivation of the *FTO* gene in mice reduced white adipose tissue mass and adipocyte size, and protected from diet induced obesity [9]. In addition to potential central effects on food intake [10], [11], FTO has been described to affect body fat mass through regulation of lipolysis [12]. *In vitro* studies and crystal structure analyses suggested a DNA/RNA demethylase function for FTO as a potential mechanism [13]–[15]. Furthermore, a recent publication identified FTO as a transcriptional coactivator enhancing the transactivation potential of CCAAT/enhancer binding proteins [16] that are master regulators of adipogenesis. Together, these findings support a functional role for *FTO* in the development of obesity on the central and/or peripheral level. So far, there are only sparse data on how metabolic factors might modulate *FTO* expression in adipose tissue.

NAMPT, also known as pre B-cell colony enhancing factor or “visfatin”, is another novel obesity related factor and has been shown to play a role in glucose homeostasis [17]. The mRNA expression and protein release of NAMPT has been reported, in part controversially, to be influenced by glucose and insulin in murine adipocytes [18] and THP-1 monocytes [19]. In addition, a differential regulation of *NAMPT* expression by dexamethasone and isoproterenol in murine 3T3-L1 adipocytes has been reported [20]. We have previously shown that NAMPT expression is higher in adipocytes compared to preadipocytes [17] and is associated with glucose metabolism [21]. However, a putative distinct modulation of *NAMPT* transcription by metabolic regulators in human preadipocytes compared to adipocytes still remains to be elucidated.

Taken together, data from the literature suggest that FTO and NAMPT represent two novel candidates potentially involved in the pathophysiology of obesity and associated metabolic disorders. The regulation of these two factors in adipocytes remains less clear. We, therefore, aimed to evaluate *FTO* expression during human adipocyte differentiation to assess whether the association of *FTO* with obesity may be directly related to adipogenesis. Furthermore, we investigated a potential transcriptional regulation of *FTO* and *NAMPT* in preadipocytes and adipocytes by metabolic regulators.

## Materials and Methods

### Cell culture of preadipocytes and adipocytes

Experiments were performed using the Simpson-Golabi-Behmel syndrome (SGBS) cell model which was a generous gift from M. Wabitsch (Ulm, Germany). Cells were cultured in basal SGBS medium consisting of DMEM/Ham F12 medium (Life Technologies, Karlsruhe, Germany) supplemented with 33 µM biotin and 17 µM pantothenic acid. Cells were differentiated into mature adipocytes as previously described [22]. Briefly, SGBS preadipocytes were grown to confluence in basal medium supplemented with 10% FCS. Adipocyte differentiation was induced under serum-free conditions by supplementing basal medium with 20 nM insulin, 0.2 nM triiodothyronine, 100 nM hydrocortisone, and 0.13 nM apo-transferrin. For the first 4 days of differentiation, 2 µM rosiglitazone, 25 nM dexamethasone and 500 µM 3-isobutyl-1-methylxanthine (IBMX) were additionally added. Cells were harvested every other day from day 0 to day 12 post-induction. Human subcutaneous preadipocytes from three male individuals (Caucasian, 21–60 years old, non-diabetic) were purchased from LONZA (Walkersville, USA) and were cultured and differentiated according to the manufacturer's instructions. Cells were harvested every other day from day 0 to day 12 post-induction.

### Stimulation with metabolic regulators

Stimulation experiments were performed in SGBS preadipocytes and mature adipocytes at day 10 post-induction under serum-free conditions. Confluent preadipocytes were serum starved for 24 h and then cultivated in SGBS basal medium supplemented with either 100 nM insulin or 100 nM dexamethasone, 100 nM IGF-1 or 10 µM isoproterenol for additional 24 h for. For stimulation experiments in adipocytes we applied two different experimental approaches: i) the adipocyte cell medium was directly replaced by basal SGBS medium containing respective stimulants (see above) for 24 h (unstarved from adipogenic supplements) and ii) adipocytes were starved from adipogenic supplements in SGBS basal medium for 24 h prior to stimulation. Untreated cells incubated in basal SGBS medium for 24 h served as control (C). For stimulation with D-glucose the cell culture medium was replaced by DMEM adjusted to 0, 2.2, 4.4, 8.8, 17.5 or 35 mM D-glucose and supplemented with 33 µM biotin and 17 µM pantothenic acid for 48 h. Preadipocytes and adipocytes cultured for 48 h in DMEM adjusted to 17.5 mM glucose, which is consistent with the glucose concentration in basal SGBS medium, served as control.

### Analysis of mRNA expression

Total RNA was extracted using the RNeasy Mini Kit (Qiagen, Hilden, Germany) according to the manufacturer's instructions. Reverse transcription was performed using 200 U M-MLV reverse transcriptase per 500 ng total RNA (Invitrogen, Karlsruhe, Germany) with random hexamer [p(dN)_6_] primers. *FTO*, *RBL2*, *NAMPT* and *peroxisome proliferator-activated receptor γ (PPARγ)* mRNA expression was quantified by *real-time* PCR with TaqMan probe-based gene expression assay on the ABI 7500 Sequence Detection System (Applied Biosystems, Darmstadt, Germany). The housekeeping genes *Hypoxanthine-guanine phosphoribosyltransferase (HPRT)*, *ß-actin (ACTB)*, and *TATA-box-binding protein (TBP)* were quantified simultaneously. Sequence information of primers and probes is given in the Table 1. For standardization of gene expression target genes were normalized to the mean of the three housekeeping genes. Basal expression of *FTO, RBL2, NAMPT and PPARγ* in preadipocytes (d0), in untreated cells (C) or in cells incubated in 17.5 mM glucose was set 1.

**Table 1:** Sequences of primer and probes.

Gene	Forward primer	Reverse primer	Probe
*FTO*	TTGGCCGGTTCACAACCTC	AGCCAACTGACAGCGTTGTAAA	TCCTGTTGAGCACTCTGCCACTCGG
*RBL2*	TTGGCATGGAAACCAGAGTCT	GCCAGCAATGCAAATTTCATC	TTCCTACATGTGAAGAGGTCATGCCACCT
*NAMPT*	GCAGAAGCCGAGTTCAACATC	TGCTTGTGTTGGGTGGATATTG	TGGCCACCGAACTCAC
*PPARγ*	GATCCAGTGGTTGCAGATTACAA	GAGGGAGTTGGAAGGCTCTTC	TGACCTGAAACTTCAAGAGTACCAAAGTGCAA
*ACTB*	TGAGCGCGGCTACAGCTT	CCTTAATGTCACGCACGATTT	ACCACCACGGCCGAGCGG
*TBP*	TTGTAAACTTGACCTAAAGACCATTGC	TTCGTGGCTCTCTTATCCTCATG	AACGCCGAATATAATCCCAAGC GGTTTG
*HPRT*	GGCAGTATAATCCAAAGATGGTCAA	GTCTGGCTTATATCCAACACTTCGT	CAAGCTTGCTGGTGAAAAGGACCCC

### Statistical analyses

Data are presented as means ± SEM of at least 3 independent cell culture experiments, each performed in triplicates. Expression during adipogenesis was analyzed applying one-way ANOVA with repeated measurements and Dunnett's post test. The differences between means in stimulation experiments were analyzed by Student *t*-test. The threshold for statistical significance was set at *P*<0.05. Statistical analyses were performed using Graph Pad Prism 4 (GraphPad Software Inc., San Diego, CA, USA).

## Results

### *FTO* expression during adipogenesis

**Figure 1:**
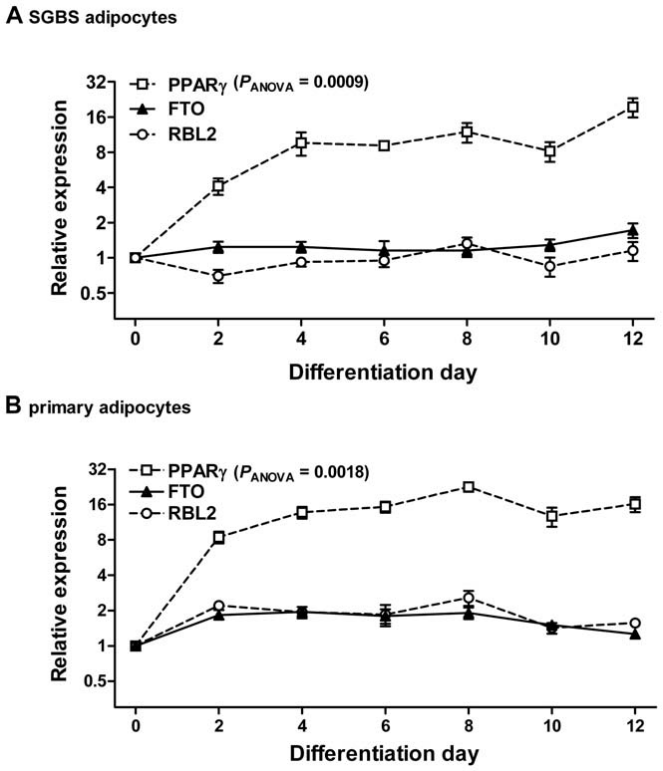
*FTO* and *RBL2* expression during adipogenesis. *FTO* and *RBL2* expression was not modulated during *in vitro* differentiation of SGBS (**A**) and primary subcutaneous (**B**) preadipocytes to mature adipocytes (expression in preadipocytes at day 0 was set = 1). An expected increase in mRNA expression of *PPARγ* confirmed efficient adipogenesis. Data are shown for at least 3 independent cell experiments. Statistical significance was assessed by ANOVA with repeated measurements and Dunnett's post test. Data are mean±SEM.

We first evaluated, whether *FTO* mRNA expression is modulated during human adipocyte differentiation. Over the differentiation period of 12 days, *FTO* mRNA levels remained unchanged in the SGBS cell model (Figure 1A) as well as in primary (pre)adipocytes (Figure 1B). The 20fold increase of *PPARγ* expression confirmed an effective adipocyte differentiation and was similar in both cell models. Hence, *FTO* mRNA expression is not significantly modulated during differentiation and maturation of human adipocytes. Similar to *FTO*, *RBL2* expression was not transcriptionally regulated during adipogenesis (Figure 1A+B).

### *FTO* expression in response to metabolic regulators

**Figure 2:**
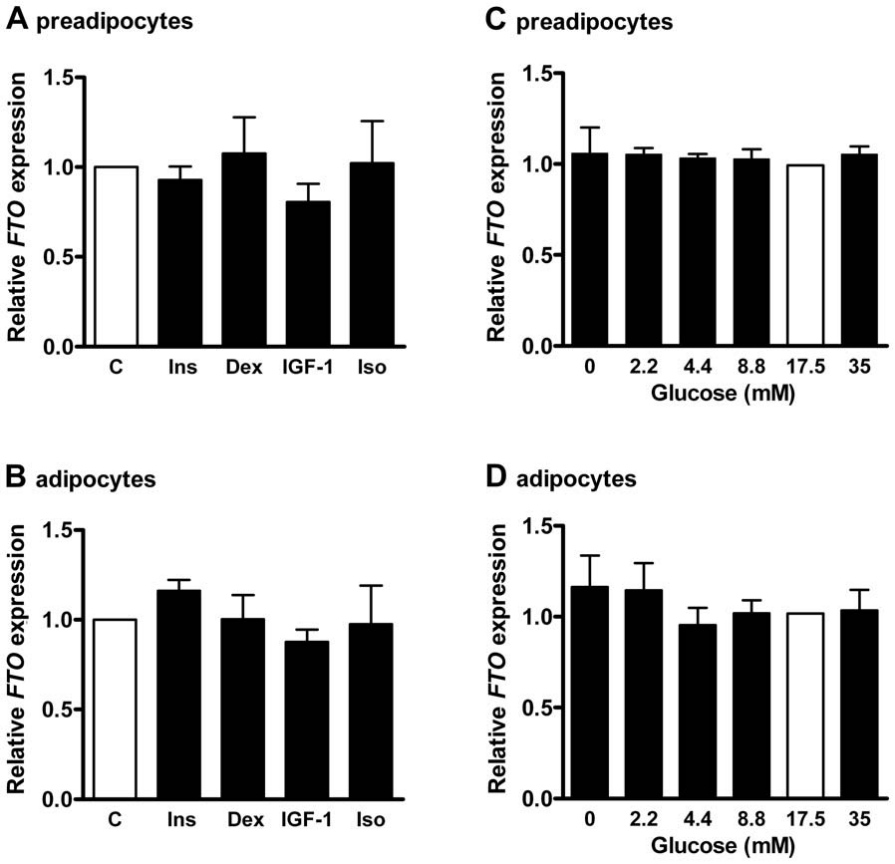
*FTO* expression in response to metabolic regulators. Preadipocytes (**A+C**) and adipocytes (day 10 of differentiation) (**B+D**) were stimulated with 100 nM insulin (Ins), 100 nM dexamethasone (Dex), 100 nM IGF-1 and 100 nM isoproterenol (Iso) for 24 h and with increasing concentrations of glucose for 48 h as indicated. Expression in untreated cells (C) and at 17.5 mM glucose was set = 1. Data are shown for 3 independent cell experiments. Statistical significance was assessed by student's t-test.

In preadipocytes and adipocytes, insulin, dexamethasone, IGF-1 and isoproterenol had no effect on *FTO* mRNA expression (Figure 2A+B). When adipocyte culture medium was not depleted from adipogenic supplements (insulin, triiodothyronine, hydrocortisone) 24 h prior to stimulation experiments, treatment of adipocytes with 100 nM IGF-1 resulted in a moderate reduction of *FTO* expression by 15.7±5.1% (*P* = 0.03), while incubation with insulin, dexamethasone and isoproterenol did not modulate *FTO* mRNA level (data not shown). Altering glucose concentrations did not affect *FTO* expression in preadipocytes or adipocytes (Figure 2C+D).

### *NAMPT* expression in response to metabolic regulators

**Figure 3:**
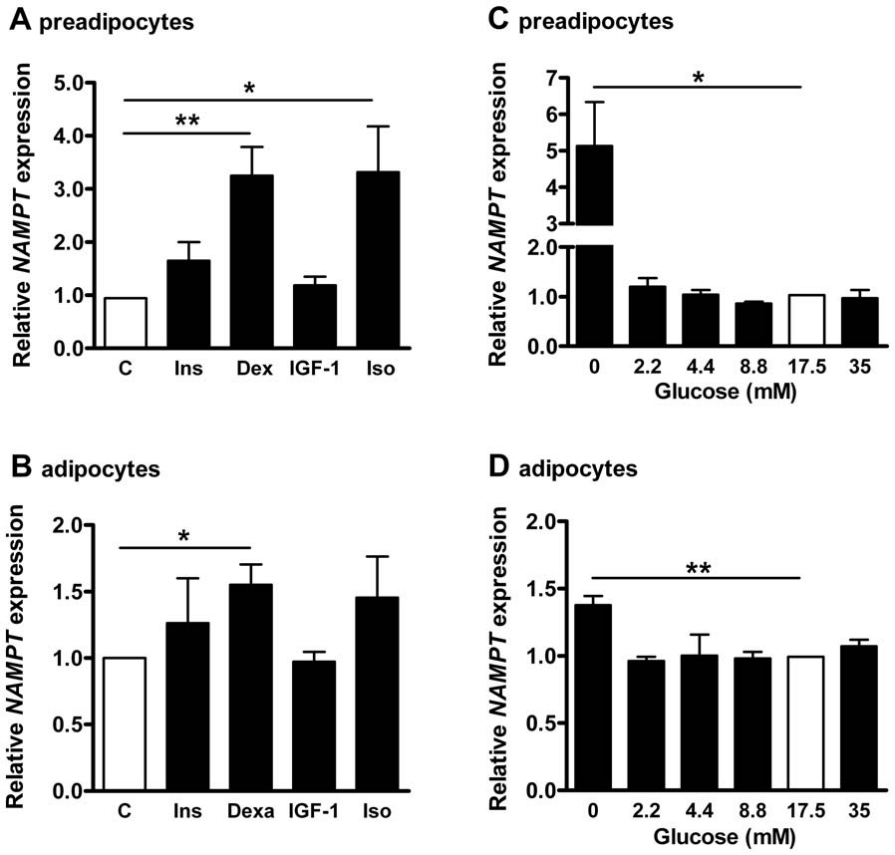
*NAMPT* expression in response metabolic regulators. Preadipocytes (**A+C**) and adipocytes (day 10 of differentiation) (**B+D**) were stimulated with 100 nM insulin (Ins), 100 nM dexamethasone (Dex), 100 nM IGF-1and 100 nM isoproterenol (Iso) for 24 h and with increasing concentrations of glucose for 48 h as indicated. Expression in untreated cells (C) and at 17.5 mM glucose was set = 1. Data are shown for 3 independent cell experiments. Statistical significance was assessed by student's t-test.

*NAMPT* expression was significantly induced 3.2±0.5 fold by dexamethasone and 3.3±0.9 fold by isoproterenol in preadipocytes, while insulin and IGF-1 did not alter *NAMPT* mRNA levels (Figure 3A). In adipocytes the stimulation effect of dexamethasone was slightly diminished to 1.6±0.2 fold (Figure 3B). In adipocytes cultured in medium that was not depleted from adipogenic supplements 24 h prior to stimulation experiments, neither insulin, IGF-1, dexamethasone, nor isoproterenol did affect *NAMPT* mRNA expression (data not shown).

Increasing glucose concentrations had no impact on *NAMPT* expression, but complete glucose restriction stimulated *NAMPT* mRNA expression by 5.1±1.2 fold and 1.4±0.1 fold in preadipocytes and adipocytes, respectively (Figure 3C+D).

## Discussion

In this study, we evaluated the potential regulation of *FTO* and *NAMPT* as two novel candidates potentially involved in the pathophysiology of obesity and metabolic sequelae by metabolic regulators at the level of adipocytes.

We show that expression of the “obesity gene” *FTO* is not significantly affected by adipocyte differentiation or metabolic regulators related to glucose and insulin metabolism in our cell models of human subcutaneous adipocytes. Variants in the *FTO* gene have been unequivocally and robustly associated with obesity risk [1], [3], [4]. We hypothesized that FTO may be related to adipogenesis and, therefore, evaluated *FTO* expression during human adipocyte differentiation. *FTO* mRNA expression was, however, not modulated during differentiation of preadipocytes into mature adipocytes suggesting that *FTO* expression in adipocytes may not be related to increasing adipose tissue mass. Also, mRNA levels of *RBL2*, that was suggested to mediate the biological consequences of a *FTO* variant on obesity risk [5], remained unchanged. An increase of *PPARγ* expression during adipogenesis confirmed efficient adipocyte differentiation, hence excluding a bias by suboptimal adipogenesis. Our results somewhat contrast the findings of previous studies demonstrating that *FTO* expression is decreased during differentiation of primary preadipocytes isolated from human subcutaneous adipose tissue [12] or SGBS preadipocytes [23]. The discrepancy might be explained by differences in experimental protocols with respect to exposure to adipogenic supplements. On the other hand, we and others provided evidence that adipocyte aging is associated with a reduction in gene expression of adiponectin, leptin and PPAR*γ* after day 10–12 [24], [25]. Hence, the reported decrease of *FTO* expression at day 14 post induction in the study of Tews et al. [23] might at least be attributed to the aging process. Nevertheless, analyses were restricted to subcutaneous adipocytes so far; it has been clearly demonstrated that subcutaneous and visceral adipocytes exhibit differences in gene expression and metabolic properties [26]. Thus, a putative differential regulation of *FTO* during adipogenesis in adipocytes obtained from other fat depots such as visceral adipose tissue still needs to be elucidated.

A potential role for *FTO* in the development of obesity was proposed by studies in mice showing that Fto deficiency resulted in reduced body weight and white adipose tissue mass [9], [27]. Complete absence of Fto was also characterized by growth retardation [9]. Considering that IGF-1 stimulates lipogenesis and that FTO has been shown to increase lipolysis [12], [28], one may speculate that IGF-1 and FTO may counteract as metabolic regulators in adipose tissue. Also, whole body and neural Fto knockout mice have reduced serum levels of IGF-1 suggesting that FTO regulates directly or indirectly IGF-1 secretion by the liver through the hypothalamus-pituitary axis [29] or as transcriptional coactivator [16] via C/EBP binding to the IGF-1 promotor [30]. Together, this may point to a putative interaction of FTO and IGF-1 in several tissues.

To further evaluate the interaction with metabolic regulators, we investigated the impact of glucose, insulin as well as dexamethasone and isoproterenol, representing exogenous compounds that affect the stress axis, on *FTO* expression in preadipocytes and adipocytes to mimic different metabolic states of energy excess and energy deficiency in preadipocytes and adipocytes. However, neither glucose or insulin nor dexamethasone or isoproterenol did affect *FTO* gene expression. Our results hence indicate that nutritional status does not influence *FTO* expression at the level of adipocytes in our models. *FTO* is widely expressed across multiple tissues including adipose tissue, liver, pancreas, and skeletal muscle. The highest abundance of *FTO* mRNA was, however, found in the brain, particularly in the hypothalamus [1], [13]. Rodent studies revealed that *FTO* expression is nutritionally regulated in these hypothalamic regions [13], [31]. Furthermore, several studies in humans [32], rats [33] and mice [9], [29] indicated that *FTO* acts on the level of the central nervous system mainly in the arcuate nucleus of hypothalamus.

Overall, these results indicate that the brain rather than adipocytes are the primary site of *FTO* regulation by nutritional status. But the regulation of *FTO* by metabolic factors in adipocytes derived from different adipose tissue depots is still elusive.

NAMPT has been found to play an important role in the regulation of glucose metabolism, primarily through effects on beta cell function [17]. In adipocytes NAMPT has also been shown to be affected by cytokines and hormones related to glucose homeostasis in murine 3T3-L1 cells [20], [34]. In our study, the compounds that affect the stress axis, namely dexamethasone and isoproterenol induced *NAMPT* expression in human preadipocytes. In line with this, dexamethasone increased *NAMPT* mRNA levels in murine 3T3-L1 adipocytes [20], [35], while isoproterenol decreased *NAMPT* expression in the murine model [20]. These controversial results may reflect differences in the cell models and/or species as has been shown for other adipocytokines [36].

Of interest, the effect of dexamethasone and isoproterenol on *NAMPT* expression was more pronounced in preadipocytes compared to adipocytes in our study. There are several possible explanations for that. First, NAMPT is mildly increased during adipogenesis [17], [20], [37] and the higher basal expression in mature adipocytes and/or the supplement of dexamethasone for the differentiation may have reduced responsiveness to the factors in mature adipocytes. Second, changes in the gene expression pattern during adipocyte differentiation [38] may also account for an altered sensitivity of preadipocytes and adipocytes. Third, preadipocytes are potentially more sensitive to distinct stress stimuli than adipocytes as shown for LPS stimulated production of cytokines such as IL-6, TNF-α, MCP-1 and IL-8 which was more pronounced in preadipocytes than in adipocytes [39], [40].

Considering that NAMPT release has been reported to be enhanced by glucose in adipocytes [18], we expected an increase of NAMPT with increasing glucose concentrations. However, high glucose concentrations did not affect *NAMPT* mRNA levels. Complete glucose restriction, on the other side, induced *NAMPT* gene expression. Again, this effect was more pronounced in preadipocytes compared to adipocytes. Also other studies have reported that *NAMPT* expression is induced by glucose restriction and fasting [41], [42]. Complete glucose deprivation induces endoplasmic reticulum (ER) stress and cell death. As a NAD biosynthetic enzyme, NAMPT is involved in the regulation of a variety of biological processes, such as differentiation, metabolism and stress response [43].We thus hypothesize that our results may reflect an adaptive response to cellular stress due to reduced nutrient availability [41], [44].

In contrast to glucose, insulin had no effect on NAMPT synthesis in our cell model, which is consistent with data from studies in murine as well as primary human adipocytes [18], [20]. Taken together, our data indicate that *NAMPT* expression in subcutaneous adipocytes is not regulated by high insulin and glucose concentrations that represent common features of insulin resistance and type 2 diabetes. Interestingly, the stress axis affecting compounds dexamethasone and isoproterenol, that are known to induce insulin resistance in adipocytes [45], [46] induced *NAMPT* transcription. Our results indicate that *NAMPT* mRNA levels are enhanced in response to cellular stress but appear to be not regulated by nutrients in human adipocytes *in vitro*.

A potential limitation of our study is that the SGBS cell model is a cell line that may not necessarily respond the same as normal adipocytes. Hence, our results on action and regulation of *FTO* and *NAMPT* can not be generalized to the level of adipose tissue.

In summary, we demonstrate that *FTO* gene expression is not related to differentiation and maturation of human adipocytes. Furthermore, we show that *FTO* expression is not regulated by factors known to impact glucose metabolism. *NAMPT* expression was induced by stress axis affecting compounds and complete glucose restriction supporting a potential role in compensation of cellular stress response in preadipocytes and adipocytes.
